# How much of my true self can i show? social adaptation in autistic women: a qualitative study

**DOI:** 10.1186/s40359-023-01192-5

**Published:** 2023-05-03

**Authors:** Mebuki Sunagawa

**Affiliations:** Department of Psychology, Ochanomizu University, 2-1-1 Otsuka, Bunkyo-ku, Tokyo 112- 8610 Japan

**Keywords:** Social adaptation, Autistic women, Adult, Qualitative study

## Abstract

**Background:**

Social adaptation is often aimed at supporting autistic people, yet its specific goals may not include their actual perspectives. That is, the state of adaptation is judged based on the standards and values of non-autistic people. This qualitative study focused on autistic women’s perceptions of social adaptation and examined their lived experiences in daily life, as adaptive behaviors have often been reported as a “female autism phenotype.”

**Methods:**

Semi-structured interviews were conducted face-to-face with ten autistic women aged 28–50 years (*M* = 36.7; standard *SD* = 7.66). The analysis was conducted based on the grounded theory approach.

**Results:**

Two core perceptions were identified: maintaining stable relationships and fulfilling social roles based on past experiences of “maladaptation.” The participants sought adaptations within a reasonable range and adjusted their balance with society to maintain stability in their daily lives.

**Conclusion:**

The findings indicated that autistic women’s perceptions of adaptation were based on the accumulation of past negative experiences. Further harmful efforts should be prevented. Support for autistic people to make their own choices in life is also important. Moreover, autistic women need a place where they can be themselves and be accepted as they are. This study showed the importance of changing the environment rather than modifying autistic people to adapt to a society.

**Supplementary Information:**

The online version contains supplementary material available at 10.1186/s40359-023-01192-5.

## Introduction

Autism spectrum disorder (ASD) or autism is a lifelong neurodevelopmental condition characterized by social communication and interaction difficulties, as well as restricted and repetitive patterns of behaviors, interests, and activities [1]. Autism is considered predominant in men. A systematic review conducted a meta-analysis of the sex ratio of autism reported in 44 population-based prevalence studies, which used criteria from after the introduction of the DSM-IV and the ICD-10, demonstrated that the overall male-to-female ratio was 4.20 (95% CI 3.84–4.60) [2]. However, studies with a lower methodological bias and that screened the general population revealed lower numbers, which may suggest that women are at risk of being overlooked, under-identified, and under-diagnosed [3]. Studies have demonstrated that women were less likely to be diagnosed or were diagnosed at a later age than men [4–6].

Social camouflaging has recently attracted attention as one of the adaptive behavioral strategies of these “unrecognized” autistic women [7]. Social camouflaging is defined as strategies that autistic people learn and use consciously or unconsciously effectively during social situations to minimize appearance of autistic characteristics and engage in society, where the majority of the population is non-autistic people [7, 8, 15]. Autistic women have reported that they pretend and hide their true selves to obtain social acceptance [9–11], by “wearing a mask,” taking on a particular “persona,” and automatically “mimicking” the person they are speaking to [9]. Similar social coping strategies had also been shown; for example, Livingston, Shah, and Happé [12] found that adults with and without a diagnosis of ASD who reported experiences of social difficulties engaged in complex and flexible “deep compensations” that facilitated social cognition, and not just a simple imitation of “shallow compensation.” While social camouflaging has adaptive aspects of coping with social situations, it also has tremendous costs, including excessive mental and physical strain and confusion of self-identity [9], anxiety and depression [13], and suicidality [14]. Furthermore, successful camouflaging may lead to barriers in accessing support and diagnosis [15], while poor camouflaging may reinforce the negative perception of oneself.

Adaptive functions of autistic people in daily life are often examined in research and clinical situations. The Vineland Adaptive Behavior Scales, Second Edition (Vineland-II) is an internationally used scale that objectively assesses the level of adaptive behavior of individuals on intellectual, mental, or developmental disabilities [16]. Vineland-II defines adaptive behavior as social skills required for everyday living to ensure personal and social satisfaction, measured using four subscales: communication, daily living, socialization, and motor skills [16]. Autistic people have been shown to have unique profiles of adaptive functioning, with scores of socialization being the most low domain of adaptive behavior, followed by communication, daily living skills, and motor skills [17]. Furthermore, a large gap was identified between IQ and adaptive functioning scores, which was related to higher levels of co-occurring mental illnesses and neurodevelopmental conditions [18]. Thus, among autistic people, cognitive functioning does not necessarily correlate with daily adaptation and mental health. Kim and Bottema-Beutel [19] demonstrated a positive association between social functioning and quality of life in autistic adults and noted the positive and negative impact of camouflaging on this relationship. Therefore, camouflaging and other adaptive strategies, particularly in autistic adults without intellectual disability, could superficially produce socially functioning and conceal that they are struggling in their daily lives and require support. As such, adaptive functioning and behavior are essential aspects closely linked to the mental health and quality of daily life of autistic people.

While social adaptation is important, and support or intervention for autistic people often aims to promote it, it is unclear what social adaptation means to them. While the adaptive functions of autistic people have been extensively investigated, the discussion is often presented from the perspective of non-autistic people, with few studies focusing on social adaptation from the perspective of autistic people themselves. It is possible that autistic people experience difficulties while maintaining superficial adaptations. It has been noted that promoting camouflaging behaviors may worsen mental health [13, 20, 21]. Although social adaptation is closely related to the lives of individuals, concept and goal setting are often defined and assessed by non-autistic people. As Milton [22] highlighted the issue of mutual understanding in the double empathy problem, imposing perspectives of non-autistic people where one does not consider the perspectives of autistic people could create discrepancies between mutual perceptions, which may result in excessive adaptation of autistic people.

Therefore, social adaptation should be examined from the perspective of autistic people. The necessity of reflecting the voices of autistic people in research has been emphasized in the previous studies [21, 23–25]. This study focused mainly on the lived experiences of women, as adaptive behaviors have often been reported as a “female autism phenotype” [9, 26, 27]. In addition, the society to which women and men adapt and the roles they play differ, autistic women experience difficulties in adaptation as social expectations of women change, especially after adolescence [11]. This study aimed to examine how autistic women perceive and deal with social adaptation.

A qualitative approach was adopted to achieve this, and data obtained from in-depth interviews were examined. Qualitative research takes the participants’ narratives and explores the way they see, understand, and experience the world from their perspective [28]. This study aims to understand the internal experiential world of autistic women and inductively explore the process and meaning from their perspective. The following research questions were posed:

RQ1: What do autistic women perceive social adaptation as, and why?

RQ2: How do autistic women deal with social adaptation in daily life?

RQ3: What support do autistic women need for social adaptation?

## Methods

### Participants

The participants were ten autistic women. The inclusion criteria were (a) having a clinical diagnosis classified as ASD, (b) being diagnosed in late adolescence or adulthood (aged 16 years or older), (c) living in Japan and being able to speak in Japanese, and (d) without intellectual disability. Confirmation of diagnosis was done by participants’ self-report. Information on the participants is presented in Table 1. The age of participants ranged from 28 to 50 years (*M* = 36.7; standard deviation [*SD*] = 7.66), and the age of diagnosis ranged from 17 to 36 years (*M* = 27.6; *SD* = 6.25). None of the participants reported a diagnosis of intellectual disability. Four participants had a diagnosis of ASD, three had Asperger’s syndrome, and the remaining three had pervasive developmental disorders. Four participants also had a diagnosis of attention-deficit/hyperactivity disorder or its traits from clinicians. Three of the participants were treated for other co-occurring psychiatric conditions at the time of the interview. Two participants were employed full-time, one was self-employed, and four were employed part-time. The remaining three were unemployed; however, one managed a self-help group, and two participated in a communication program for autistic people at the hospital. All participants had some work experiences. Four participants were married, and six were single. Three participants lived independently, four lived with their spouses and children, one was in a group home, and two lived with their family of origin. Five participants graduated from university, three participants graduated high school, and one participant graduated junior college.

**Table 1:** Characteristics of the sample participants

Case number	Age	Age at ASD diagnosis	Diagnosis^a^	Additional diagnoses/ psychiatric symptoms	Employment status	Living arrangements
1	41	36	Asperger’s syndrome, ADHD	(Depression, PTSD, social anxiety disorder, hyperventilation syndrome)^b^	Part-time	With a partner and a dependent
2	40	30	Pervasive Developmental Disorder	(Bipolar disorder)	Self-employment	With a partner and a dependent
3	50	36	Pervasive Developmental Disorder	nil	Part-time	With a partner and dependents
4^c^	48	35	Asperger’s syndrome	(Bipolar disorder)	Part-time	With a partner and other family member(s)
5	38	25	Asperger’s syndrome	nil	Unemployed	Self-reliant
6	34	24	Pervasive Developmental Disorder	nil	Full-time	Self-reliant
7	31	21	ASD, ADHD	(Depression)	Part-time	In supported accommodation
8	29	17	ASD	Delusional disorder	Unemployed	Self-reliant
9	28	24	ASD, ADHD	Panic disorder	Unemployed	With parents
10^c^	28	28	ASD, ADHD	Obsessive-compulsive disorder	Full-time	Self-reliant

ASD = Autism spectrum disorder; ADHD = Attention-deficit/hyperactivity disorder; PTSD = Post traumatic stress disorder

^a^ Self-reported diagnostic name by the participant.

^b^ ( ): disorders and symptoms that are currently under control.

^c^ Participated in follow-up interview.

### Procedure

The researcher was engaged in an autistic adult self-help group as a non-autistic staff member. Participants were contacted via a support center for autistic people, self-help groups, and online networks and recruited using snowball sampling. All referred participants were made aware that the researcher was a university-affiliated woman who conducted research with autistic women. This was done to build trust among the participants for the interview, since most of them were participating in research for the first time. Interested participants were asked to contact the researcher or contacted by the researcher through contacts, provided a brief exploration of the study purpose, methodology, and ethics, and requested to confirm that they met the inclusion criteria. The researcher answered questions from participants as clearly as possible during the prior e-mail correspondence, before and after the interview. All participants provided written informed consent prior to the study. The interviews were conducted between September 2018 and February 2019. Ethical approval for this study was obtained by the Tohoku University Ethics Committee (IRB 2018-002).

### Data collection

Semi-structured interviews were conducted face-to-face with an interview guide. To build rapport and comfort with the participants, the researcher conducted a conversation on daily topics. At the beginning of the interview, participants were asked about demographic factors, including age, diagnosis, occupation, and related topics. Subsequent questions explored the participants’ experiences and perceptions of social adaptation. The interview contained questions in the following categories: (a) experiences before diagnosis; (b) experiences after diagnosis until the present; and (c) social adaptation, such as, “What do you think social adaptation is about?,” “Do you think you are adapted to society?,” and “Is there anything you do to adapt to society?” The detailed interview schedule can be found in Additional File 1. The interviews were 54–94 min long (*M* = 71.40; *SD* = 12.05). After the initial analysis, some participants were invited for additional interviews to check the provisional results to confirm their validity and suitability and provide further information. Two participants (participants 4 and 10) took part in follow-up interviews via an online video conferencing system. The follow-up interviews were 94 and 97 min. In the follow-up interviews, additional questions were asked regarding (a) their perception of current social adaptation condition and the reasons for it and (b) their stance on how to maintain a balance with society, as at the time of initial analysis, it was considered that there were differences among the participants. All interviews were audio-recorded using a digital voice recorder with permission from the participants, and verbatim recordings were made. People’s name were anonymized assigning letters of the alphabet, information that was less important from an analytical perspective, such as place, company name, and school names were removed from the transcripts.

### Data analysis

The analysis was conducted based on the grounded theory (GT) approach [29], which aims at theory generation based on data and examines the process of formation. The existing phenomenon of social adaptation is understood within the framework of non-autistic people. However, the researcher was interested in autistic women’s perspective and description of the world, as well as their perceptions, backgrounds, and responses to society, especially the processes involved therein. Thus, GT was considered appropriate for this study. Because the original GT method is a demanding procedure that has noted to be difficult to complete [28] in such small qualitative research, the following analysis was conformed to the method.

First, all verbatim recordings were read thoroughly. Subsequently, the entire data set was divided into smaller parts and given labels that reflected the characteristics of each section. Later, the labels that were thought to be related were collected, and their relationships were examined to create categories. The relationships between the assumed categories were compared with the data to confirm their appropriateness, and the labels and category names were revised as necessary. After categories were obtained through the above process, the relationships between each category were examined, and category groups containing multiple categories were generated. For clarification, the categories created in the second step are referred to as subcategories, while groups containing several subcategories are referred to as categories. Data were analyzed using MAXQDA 2020. The categories and subcategories are presented in Table 2. The storylines related to the grounded theory from this study can be found in Additional File 2 and the model diagram in Additional File 3. The researcher was interested in how autistic people experience and live in the world and wanted to “learn” and “know” from the real voices of such people. Based on the counseling skills acquired as a psychologist, the researcher tried to listen to participants’ own words without value judgments as much as possible, but the desire to explore their unique experiences might have affected the researcher’s attitude and choice of words. During these data collection and analysis processes, the researcher referred back to the interactions between the interviewer and interviewee in the raw data to check whether certain words were used by the interviewee herself or came from the researcher’s statements, and excluded inductive interactions from the analysis.

**Table 2:** Categories and subcategories

Category	Subcategory
Maintaining relationships with other people	Having a social relationship Be in a balanced relationship Maintain harmony with other people
Fulfilling one’s role	Have a job and being independent Fulfilling one’s social role
Past experiences of “maladaptation”	Difficulties in interpersonal relationships Difficulties at work
Trying to adapt to society	Expectations and evaluations of others Learning from previous social experiences
Trying to live as I am	Difficulties due to characteristics Understanding and coping with my characteristics
Coming to terms with society	State of my social adaptation What I keep in mind regarding social adaptation Understanding and acceptance from the environment

## Results

### RQ1: what do autistic women perceive social adaptation as, and why?

#### Maintaining relationships with other people: having a social relationship

Most participants mentioned that having a connection with other people, through employment and in the community, with friends, or during leisure time, and not being in a state of social withdrawal was an essential aspect of social adaptation.

Some participants felt that they could not avoid relating to other people to live in society, with responses such as “People can’t live alone, we have to interact with other people” (participant 5) and “It’s difficult, but in order to work and study, I have to interact with people” (participant 7).

Many participants were motivated to relate to other people, such as one participant who expressed her desire to have close friends: “I want to have a friend that I can talk to about absolutely ordinary things when we meet” (participant 10). The reasons participants sought interpersonal relationships were not only positive but also passive. Considering their characteristics, some participants predicted that if they withdrew from social relationships, their condition would deteriorate. One participant stated:

*If I were to stay indoors and do whatever I wanted without adapting to society, I would dwell deeper into it, not be able to see people anymore, and probably end up doing and eating whatever I wanted. Finally, I think I would end up being socially withdrawn* (participant 6).

#### Maintaining relationships with other people: be in a balanced relationship

In many cases, participants had been in relationships with overwhelming disadvantages; therefore, they noted the need for stable individual balance with mutual respect and benefit and without mutual strain. One participant emphasized that assertive communication and being able to express one’s true thoughts in a relationship wherein both parties respect each other would be ideal:

*There are a lot of things that I cannot say because I feel inferior, being an autistic person. Hence, I think it is important that I can tell people what I want to say. I think that is a large part of it. It is not just being told what to do; I mean, it is an equal relationship* (participant 4).

Participant 5 mentioned that relationships should be mutually beneficial, stating, “To control one’s power in response to what the other person is asking for is one way of adaptation.” As she could not understand what the other person required, she made sacrifices without limits. Based on such self-sacrificing experiences in the past, she emphasized reciprocity in personal relationships.

Furthermore, as a result of maintaining a good balance between people, participants thought that everyday life without being effected by severe mental or physical disruptions was crucial to determining adaption. Participant 2 stressed the importance of “building stable relationships in the community or the workplace without becoming depressed.”

#### Maintaining relationships with other people: maintain harmony with other people

Participants frequently noted that reading the atmosphere, following rules, and being considerate of others were required for social adaptation. Participant 10 stated that “the ability to work with others in a cooperative and harmonious way without ruining the atmosphere was required.”

Furthermore, reading the atmosphere differently often had negative consequences. Participant 9 noted, “In a society with detailed rules, whether you can remember to follow those rules or not is important.” As she was unaware of the rules in the past, she was bullied. She stated that “being bullied meant being excluded, so I did not adapt to society at all.”

Many participants emphasized the importance of not making others feel uncomfortable, not disturbing the atmosphere of the situation, and not causing misunderstandings, which was often based on past experiences of “failure”.

#### Fulfilling one’s role: have a job and being independent

Some participants stated that independence, including an income from work and managing one’s own life, was vital to social adaptation. Participant 9 stated, “In the end, the most important thing for a person to be considered socially well-adapted is to have a certain amount of money and to be able to live independently.”

The participants highlighted various aspects of the importance of having a job, such as economic reasons, self-image (e.g., “The image of social adaptation is working” [participant 8]), and contribution to society. Participant 7 explained the reason she emphasized work as a process of elimination, stating, “If you are not performing well enough at your work, but you can develop relationships with people smoothly, then you can get along. I am not very good at the latter.”

#### Fulfilling one’s role: fulfilling one’s social role

Participants’ responses regarding social roles varied, including work, family, school, and community. Participant 10 stated, “I think that being useful to others in a way possible, fulfilling some kind of role is a way of adapting to society. […] cooking or doing household chores for the family is an important role too.”

The participants’ living conditions, including those who were married, had children, and worked, varied, and the perception of social roles were not necessarily dependent on their current situation. Participant 5 stated that fulfilling social roles led to finding her place in society: “Social adaptation is after all about how to create a balance between society and perhaps finding a place where you fit in. […] I am probably trying to find a place that accepts me now.”

#### Past experiences of “maladaptation”: difficulties in interpersonal relationships

All participants talked about their sense of discomfort in interacting with others since childhood and problems in maintaining friendships. Moreover, although they seemed to be part of a group on the surface, they felt that they could not keep up with others, and it was often difficult for them to build close relationships. Nevertheless, many participants tried to fit in due to perceived obligation. Participant 4 noted, “I think that ‘I have to act perfectly’ as self-defense. I want people to think that I am a person with neurotypical development and not strange.”

Furthermore, about half of the participants were bullied in school, which had a negative impact on their perceptions of themselves and their surroundings and how they dealt with interpersonal relationships.

#### Past experiences of “maladaptation”: difficulties at work

Difficulties at work, such as making mistakes repeatedly, not doing things as others around them do, or differences in learning time and pace of work, may frustrate the people around them. The participants’ work difficulties led to deteriorating relationships in the workplace, and repeated reprimands led to mental health problems. Furthermore, even though they worked hard and felt good at work, the accumulated stress affected their physical and mental health. As a result, the participants repeatedly changed jobs due to physical and mental illness, and some experienced social withdrawal. Participant 1 stated, “When I got used to it, and the workload or the responsibilities increased, I would have a hyperventilation attack. Hence, I would quit the job and start a new job again after taking some time for medical treatment. It is a repetitive pattern.”

Participants described the accumulation of past hardships and challenging experiences as “maladaptation,” while referring to adaptation as “what I was lacking” (participant 10), “where I can cut back to get along with others” (participant 8), and “longing” (participant 2). Thus, their perceptions of adaptation were strongly influenced by what they felt they wanted but were difficult to do, based on their past experiences. Particularly before diagnosis, they believed that they could not adapt at all.

### RQ2: how do autistic women deal with social adaptation in daily life?

#### Trying to adapt to society: expectations and evaluations of others

Social expectations and evaluations were often referred to when participants mentioned the necessity to adapt socially. Particularly with regard to employment, participants indicated the direct and indirect social expectations from the surroundings, including being “worried about what other people think” (participant 10), people “talking behind my back” (participant 3), “oppression from others” (participant 5), and “people’s duty as stipulated in the Constitution” (participant 10). Participant 9 reported significant pressure to work, stating, “If I do not earn money, or at least become independent, I will be excluded; hence, I feel the pressure to adapt in order to protect myself and my family.”

In addition, some participants felt that social roles or expectations for women created difficulties in adaptation. Participant 4 noted, “When I was in really bad health, I could not cook at all, so the expectation that women should cook [was] hard for me.” Participant 10 stated, “As a woman, there are some expectations to read the atmosphere and not be too disruptive in the social situation or something like that. However, I do not think I can do that well enough.”

#### Trying to adapt to society: learning from previous social experiences

Some participants reported that what they had experienced in society affected their perception of social adaptation. Two participants worked in the welfare field, supporting people with disabilities. One of them stated, “I have told users that it is better to adapt to society while working, and I have seen a lot of situations where I really felt that way” (participant 6). Through work experiences, they gained knowledge of developmental disabilities and social security systems and saw people with various conditions that broadened their horizons.

In addition, participants gained insights from their social experiences. Participant 5 reported learning that a relationship can be formed as long as the work was done at her part-time job. On the other hand, while at school, she experienced complex interpersonal difficulties, such as rejection and isolation. Participant 2 said that the experiences of being a homemaker and raising children had changed her thinking. Previously, she had thought that if she did not earn money, she was not working or being “useful”; however, she began to believe that homemakers undertake household chores as their work.

#### Trying to live as I am: difficulties due to characteristics

Although the participants felt and even appeared to have adapted to society, they were also aware of difficulties in their daily life and required a certain amount of effort to live in the society. Participant 10 stated, “Certainly I got a diagnosis, but it does not change that I still have to work hard and do my best.”

In addition, participants were susceptible to the effects of their physical and mental health conditions, finding it challenging to go out and do everyday things when feeling unwell.

*I experienced a time when it was difficult to even go to places with people. In that sense, greeting people may be quite easy for neurotypical people, but we have to work very hard for it* (participant 1).

The autistic core characteristics as well as physical and mental health conditions had a significant impact on the participants’ state of adaptation.

#### Trying to live as I am: understanding and coping with my characteristics

All participants were diagnosed in adulthood, except for one diagnosed in late adolescence. Before diagnosis, they did not understand the difficulties they were experiencing and attempted to adapt with many “failures”. After the diagnosis, they came to understand their characteristics and learned how to deal with them, giving them some measure of control. Participant 2 stated, “After I started learning about autism and taking strategies to deal with it, I felt like my health was not so good but stable.”

Some participants said they realized that they had been pushing themselves too hard to adapt before diagnosis. However, after diagnosis, they were able to understand their characteristics and deal with social situations from a different perspective.

*I did not have confidence in myself, so I thought that if I did not work hard, I would fail as a human being. However, I gradually felt that the rules were not universal in the world or absolute, so I thought I could withdraw or stop when needed* (participant 6).

#### Coming to terms with society: state of my social adaptation

This subcategory indicated the degree of evaluation of the participants’ adaptation and reason. If the participants worked or lived independently, it positively affected their judgment of the state of adaptation. Participant 6 noted, “In my case, other people would think that I am well adapted to society because I am doing a fairly regular job.” Furthermore, some participants described current work or life as “fun,” “comfortable,” and “easy-going.” They described doing well in their current living environment without as many difficulties or problems as in the past, which was associated with a positive evaluation.

In terms of relationships, one participant said she could feel that not having “to force herself” allowed her to adapt to the current situation. Participant 4 stated:

*I feel much more at ease and less defensive when I am around neurotypical people. Moreover, I do not have to exert myself in such a weird way. That is why I think I have been able to adapt socially; well, be more natural.*

In contrast, some participants believed that they did not adapt well socially, as the current situation was far from their ideal state of adaptation. Participant 2 stated:

*If you are an average person who is well adapted to society, you wake up every morning, go to work, get along well with the people at work, and even if things do not go well, you manage to get by. […] When I compare myself to such people, I feel that I am not able to adapt at all.*

#### Coming to terms with society: what I keep in mind regarding social adaptation

All participants referred to what they were conscious of or made an effort to adapt socially in everyday life. Some participants emphasized the importance of following basic social manners, such as being punctual, keeping promises, getting up properly, and greeting people, which they learned through being reprimanded by others, self-awareness, and painful experiences of not being treated courteously by other people. Furthermore, many participants believed that their current condition was the result of continuous effort and learning conducted to cover their autistic characteristics. “I am probably not very good at socializing with people, but I have learned a lot about talking” (participant 9). “The small accumulation of the past experiences made me think that it would be wise not to talk at all” (participant 3).

In addition to countermeasures to their surroundings, participants emphasized the importance of maintaining stability in their lives. Participant 2 mentioned that she purposely limited interpersonal relationships and worked to prioritize stability in everyday life, although ideally, she wanted better: “I keep telling myself to be content with the current situation.” Likewise, other participants shared the importance of consciously managing their daily lives to reduce burden. Participant 4 stated:

*In addition to avoiding the things I was not good at, I also focused on controlling my daily energy usage, like the amount of power consumption of machines, I just focused on controlling that. […] It is hard to control emotions. […] What we can control the most is our schedule.*

The reason for such conscious adjustment in every aspect of their life was that in the past, they had overstrained themselves and suffered, forcing them to become conscious of maintaining stability within the limits of their characteristics and capacities.

#### Coming to terms with society: understanding and acceptance from the environment

Participants often experienced being avoided or reprimanded by others and had significant feelings of self-denial. They felt that no one would help them or accept them. However, some participants had experiences of being accepted and acknowledged by the people around them, which influenced their self-evaluation. According to participant 8, “I probably have developmental, social, and communication difficulties, but the people around me now accept that, to the extent that I do not feel inferior because of my autistic characteristics.”

Furthermore, the experience of being understood and accepted by the people around them helped the participants overcome their negative beliefs and gain new insights to begin adapting to society by choice rather than force. Participant 10 noted, “It is probably a really ordinary thing, but when I say something and the other person responds to what I say, and I realize that it is surprisingly interesting. That is what made me try to face [conversations with people].”

## Discussion

Although social adaptation is often aimed at supporting autistic people, the specific goals may not include the perspectives of people themselves, with the state of adaptation judged based on the standards and values of non-autistic people. This qualitative study conducted interviews with autistic women to explore perceptions regarding social adaptation among the participants. The results revealed two core perceptions of social adaptation: maintaining stable relationships and fulfilling social roles.

### Perceptions of social adaptation

The participants’ perceptions were based on the accumulation of past negative experiences of “failure”, difficulties, and denial due to their autistic characteristics, which were regarded as “maladaptation,” while social adaptation was viewed as the complete opposite state. The participants reported being socially motivated to interact with others, being helpful, and having expectations regarding social roles, which was in line with adaptive behavior, such as camouflaging or masking, as observed in previous studies [8, 30–32]. In addition, the participants reported having hope that relationships would be reciprocal and provide mental stability based on past painful experiences. Assumptions regarding social roles were not limited to work but included family and community, which was in line with previous studies indicating that this may be due to women’s diverse roles in social contexts and pressure regarding role expectations [7, 21, 33].

However, the autistic characteristics of cognitive flexibility [34] may affect the perceptions by reinforcing the idea of what adaptation should be. Non-autistic people may not always be “perfectly adapted,” some are moderately adapting by cutting corners—for example, by prioritizing things, relying on others, and maintaining a balance between work and leisure—while others may have difficulties adapting. In other words, the condition of social adaptation is just as divers among non-autistic people. In contrast, autistic people face difficulties in adjusting to the intensity of adaptive behavior, and their low self-esteem or less interactions with other people may lead to high standards of adaptation, as people around them look like they are doing well. Some participants indicated that they made excessive efforts to fit in, as they felt that they had to “work hard” and “hide their autistic characteristics.” Furthermore, as a positive association between the perceived autism stigma and camouflaging behaviors was demonstrated [35], the perceptions of social adaptation might reflect, consciously or unconsciously, the values and beliefs of society, which often did not match the participants’ characteristics and constrained them enormously. Research on the cognitive aspects of social adaptation of autistic people, such as unconscious biases or assumptions, will be helpful to understand the context of adaptive behavior in the future.

Interestingly, one participant said, “After I tried not to aim for social adaptation, I started to feel more comfortable and was able to adapt to society as a result” (participant 4). Therefore, in some cases, participants were able to adapt to society in their individual way by not forcing themselves to do so. Similar trends were identified in previous qualitative research, in which the participants demonstrated “authentic autistic socializing,” reflecting reduced desire to conform to non-autistic norms or behave in a way that appears non-autistic and engaged in living with the self as it is [31]. Livingston et al. [12] indicated that some participants adopted balanced compensatory strategies that emphasized seeking a less stressful environment rather than learning social skills to adapt to a non-autistic world. As Allely [36] noted the need to reduce rather than promote camouflaging behavior, it is essential to support autistic people to choose and manage their lives happily, comfortably, and naturally.

### Adaptation in daily life

As the participants had varied perceptions of adaptation, they tried to maintain stability in their daily lives by considering their characteristics, seeking adaptation within a reasonable range, and adjusting their balance with society. In line with previous studies [31, 37], this adaptation to maintain daily stability requires a certain degree of self-understanding. Leedham, Thompson, Smith, and Freeth [38] reported increased self-acceptance and a sense of agency after diagnosis among autistic women who were diagnosed in their midlife. The results highlighted the importance of understanding one’s strengths and weaknesses, preventing or avoiding adaptations that the participants felt negative toward, such as schedules, work, and relationships can help reduce damaging and fruitless efforts and maintain long-term stability. Some participants tried to adapt blindly to society and were unsuccessful before diagnosis; however, by learning about their characteristics after diagnosis, they were able to adopt appropriate countermeasures and choose when to adapt. In addition, the results revealed the importance of maintaining stability without excessive effort, as the physical condition affected daily life.

However, it is important to note that the process of self-understanding is challenging and painful [38]. The degree and characteristics of autistic traits vary significantly from person to person, requiring individual support. A proper understanding of autistic women and the training of practitioners is essential. While most participants noted the necessity of accurate diagnosis for social adaptation, some reported difficulties related to diagnosis, such as misdiagnosis or difficulties in getting an appointment. Furthermore, some participants indicated a lack of understanding from those around them because they did not fit the typical autistic image. Previous studies reported a similar lack of understanding and support for autistic women among professionals [9, 12, 38, 39], highlighting a need for better understanding that is not bound by existing male-based standards and understandings [40].

This study indicated that the experiences of being accepted, acknowledged, and being successful led the participants to change the way they dealt with society and their surroundings. Many participants had negative experiences of being rejected or avoided in their interpersonal relationships, leading to low self-esteem and a strong feeling of not being accepted by others, which was described as “disempowerment” in the process of diagnosis by Leedham et al. [38]. However, some participants demonstrated that positive experiences in social situations might provide new insights after initial “failures”. A previous study [41] that interviewed adolescent autistic girls indicated that when the participants felt accepted in supportive friendships, they felt freer to be their authentic selves with less need to mask themselves. Further research is needed to explore what conditions or states allow autistic people to be their authentic selves.

### Is society an obstacle to autistic people?

One participant stated that it is not the autistic people who are the obstacle, but the society that is the obstacle for them to live. This raises the question of whether it is appropriate to place responsibility for the social adaptation solely on autistic people. Discrepancies can arise between the autistic and non-autistic sides due to a lack of mutual understanding [22], and in many cases, autistic people are trying to learn and adapt to the mainstream society: non-autistic people’s culture. It has been pointed out that traditional interventions, such as modifying autistic behavior to fit social situations, can be damaging [20, 24, 31]. The importance of intervening through the environment to improve a person and social environmental fit, rather than modifying the person, has been proposed [19, 42].

This study indicated that autistic people should not be forced to unilaterally adapt to a society dominated by non-autistic people; instead, the non-autistic side should also adapt to autistic people and aim for mutual understanding and respect of diversity [31]. In other words, it is important to create a social environment where people are not forced to be “like everyone else,” where various ways of working and living are accepted, and having autistic characteristics does not become an obstacle.

Furthermore, social barriers for a disabled person are closely related to the cultural background. The perceptions of social adaptation in this study were also likely related to the sociocultural environment [43], such as the unique Japanese culture with an emphasis on harmony and peer pressure, and social conditions, such as many women being involved in non-regular employment. Furthermore, “over-adaptation,” the attempt to adapt externally at the expense of internal adaptation, is a concept unique in Japan, and in recent years, similarities with the adaptive behavior of autistic people have also been noted [44]. In the Japanese culture, it is considered adaptive to go along with the crowd to some extent, and in many cases, the person may feel secure in this way. However, such sociocultural influences were not sufficiently examined. A previous qualitative study [45] indicated that Japanese adolescent autistic girls used self-sacrificing, interpersonal, and interdependence coping strategies, which may reflect the Eastern culture of prioritizing other people’s needs in contrast to Western culture, where coping strategies were selected based on individual needs. In addition, sociocultural influences on camouflage have been noted [15]. Future studies should clarify the differences and similarities between cultures, values, manners, and other backgrounds of social adaptation.

### Study limitations

This study focused on the perceptions of autistic women involved in social adaptation and examined their lived experiences in daily life rather than social situations. However, this study had some limitations. First, the factors that may affect adaptation, such as age, living conditions, and intellectual conditions, were not controlled. Particularly, the participants’ age range was wide. Their diagnosis and support experiences, knowledge of neurodevelopmental conditions, and challenges faced at various life stages varied by age. These might have influenced the differences in their needs and stance on adapting to society. In addition, as all the participants had, to some extent, already adapted to society; those struggling with adaptation were not included in this study. Therefore, examining the experiences and values or perceptions of social adaptation among autistic women of different age groups (e.g., adolescent women, young adult women, and middle-aged women) is required.

### Implications

Social adaptation indicates leading an individually satisfying life. Ignoring individual needs may enforce a perception of adaptation that is socially correct or assumed by surrounding people. Based on the findings of this qualitative study, several important implications emerged to support healthy social adaptation in autistic people. First, their past efforts and hardships must be considered with respect, and further harmful efforts should be prevented. Support for reflecting on the past that autistic people themselves had tried to adapt excessively by internalizing the values or expectations of society, and encouraging them to realize that they do not have to push themselves to fit in or can avoid things they are uncomfortable with. Second, it is important to support them in making their choices in life rather than forcing them to adapt to society. In some cases, this may include the individual’s choice to adapt to the non-autistic surroundings; support provided to autistic people should consider their perspectives and desires. Third, positive social experiences, such as work and interpersonal relationships, may be the catalyst for negative and defensive perceptions of society among autistic people. Therefore, autistic people require an environment and understanding that does not deny their characteristics, but rather enables them to gain positive social experiences without anxiety. In addition, to improve their self-esteem, it is important to encourage them to work on what they like and are good at, rather than what they are not good at or do not like. Finally, for women in particular, in addition to having autistic characteristics, being a woman can cause difficulties in social adaptation. They are often seemingly well-adapted to society, and conceal that they are desperately trying to maintain this superficial adaptation. They need a place where they can be themselves and be accepted as they are.

## Electronic supplementary material

Below is the link to the electronic supplementary material.

Supplementary Material 1

Supplementary Material 2

Supplementary Material 3

Supplementary Material 4

## Data Availability

The datasets of the interviews are not publicly available due to privacy related issues. Information on transcripts is available from the corresponding author on reasonable request.

## Abbreviations

ASD: Autism spectrum disorder.
Vineland-II: Vineland Adaptive Behavior Scales, Second Edition.
