# Supplementary figures and images for: How much of my true self can i show? social adaptation in autistic women: a qualitative study
Source: BMC Psychol. 2023 May 3;11:144. doi: 10.1186/s40359-023-01192-5 (PMC10155366; doi:10.1186/s40359-023-01192-5)

## Slide 1
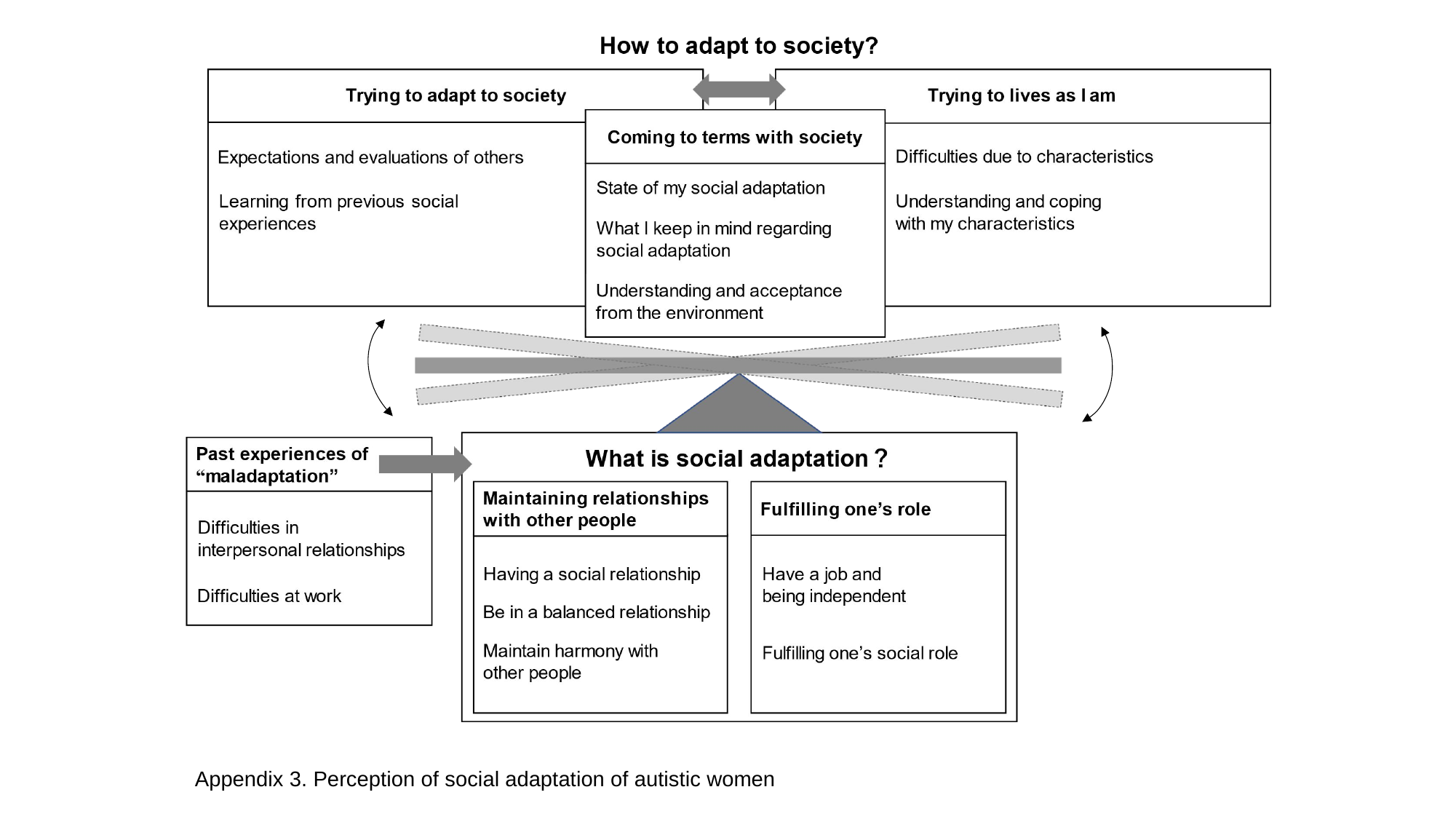

Appendix 3. Perception of social adaptation of autistic women

Supplement: Supplementary file 1 — Supplementary Material 1 [file 40359_2023_1192_MOESM1_ESM.pptx]
