# Supplementary material for: How much of my true self can i show? social adaptation in autistic women: a qualitative study
Source: BMC Psychol. 2023 May 3;11:144. doi: 10.1186/s40359-023-01192-5 (PMC10155366; doi:10.1186/s40359-023-01192-5)
Supplement: Supplementary file 2 — Supplementary Material 2 [file 40359_2023_1192_MOESM2_ESM.docx]

Appendix 1. Interview schedule

1. Tell me about yourself.

(age, diagnosis, age at diagnosis, co-occurring condition, medication, current living situation, residence, current services received, last education, occupation, hobbies etc.)

2. Tell me about the process you experienced to be diagnosed.

3. What do you think social adaptation is about?

4. Why do you think so?

5. When did you come to think so?

6. Has there been any change in your perception from the above to the present?

(If so, why?)

7. Do you think you are adapting to society?

8. Why do you think so?

9. What are the positive aspects of adapting to society?

10. What are the negative aspects of not adapting to society?

11. Is there anything you do to adapt to society?

12. When and under what circumstances do you do it? Is there any situation in which you don't do it?

13. Why do you do it?

14. Is it easy or difficult for you?

15. How did you learn to do it?

16. What support do you needed to adapt to society?

17. What do you think about your autism?
