# Supplementary material for: How much of my true self can i show? social adaptation in autistic women: a qualitative study
Source: BMC Psychol. 2023 May 3;11:144. doi: 10.1186/s40359-023-01192-5 (PMC10155366; doi:10.1186/s40359-023-01192-5)
Supplement: Supplementary file 3 — Supplementary Material 3 [file 40359_2023_1192_MOESM3_ESM.docx]

Appendix 2. Storyline*

The participants had two core perceptions of social adaptation: *maintaining relationships with other people* and *fulfilling one’s role*, which were based on *past experiences of “maladaptation,”* as participants expressed, in their interpersonal lives and at work. *Maintaining relationships* is about the condition of relationships includes in the categories of *having a social relationship, be in a balanced relationship* and *maintain harmony with other people. Fulfilling one’s role* is about how to be in society that includes categories of *have a job and being independent* and *fulfilling one’s social role*. This perception affected actual social life. In their everyday lives, the participants tried to balance their efforts to adapt to society with what they wanted to or could do. *Trying to adapt to society* was a way of living that was influenced by the *expectations and evaluations of others* and *learning from previous social experiences.* In contrast, *trying to live as I am* is a way of living that aims to maintain stability in daily life rather than the ideal with social adaptation, which shows that the participant was aware of *difficulties due to characteristics*, and tried to *understand and cope with my characteristics*.

The participants adjusted their ways of *coming to terms with society,* which included *state of my social adaptation,* *what I keep in mind regarding social adaptation, understanding and acceptance from the environment*, which varied from person to person.

* Category names are in italics.
