# Supplementary material for: How much of my true self can i show? social adaptation in autistic women: a qualitative study
Source: BMC Psychol. 2023 May 3;11:144. doi: 10.1186/s40359-023-01192-5 (PMC10155366; doi:10.1186/s40359-023-01192-5)
Supplement: Supplementary file 4 — Supplementary Material 4 [file 40359_2023_1192_MOESM4_ESM.docx]

**Additional files list**

File name: Appendix 1.

File format: .docx

Title of data: Interview schedule

Description of data: List of questions used in the interview.

File name: Appendix 2.

File format: .docx

Title of data: Storyline

Description of data: The brief description of the results of the analysis with categories from the data set.

File name: Appendix 3.

File format: .pptx

Title of data: Perception of social adaptation of autistic women (Model diagram)

Description of data: Diagrammatic depiction of the results of the analysis.
